# Value of statistical life year in extreme poverty: a randomized experiment of measurement methods in rural Burkina Faso

**DOI:** 10.1186/s12963-021-00275-y

**Published:** 2021-11-17

**Authors:** Stefan T. Trautmann, Yilong Xu, Christian König-Kersting, Bryan N. Patenaude, Guy Harling, Ali Sié, Till Bärnighausen

**Affiliations:** 1 Department of Economics, Heidelberg University, Heidelberg, Germany; 2 Utrecht School of Economics, Utrecht University, Utrecht, The Netherlands; 3 Department of Banking and Finance, University of Innsbruck, Innsbruck, Austria; 4 Department of International Health, Johns Hopkins Bloomberg School of Public Health, Baltimore, MD USA; 5 Institute for Global Health, University College London, London, UK; 6 Africa Health Research Institute, Durban, KwaZulu-Natal South Africa; 7 Centre de Recherche en Santé de Nouna, Institut National de Santé Publique, Nouna, Burkina Faso; 8 Heidelberg Institute of Global Health, Medical Faculty and University Hospital, Heidelberg University, Heidelberg, Germany; 9 Center for Population and Development Studies, Harvard University, Cambridge, MA USA

**Keywords:** Value of statistical life year, Health risks, Cost-effectiveness, Payment cards, Price lists, Extreme poverty, Burkina Faso

## Abstract

**Background:**

Value of a Statistical Life Year (VSLY) provides an important economic measure of an individual’s trade-off between health risks and other consumption, and is a widely used policy parameter. Measuring VSLY is complex though, especially in low-income and low-literacy communities.

**Methods:**

Using a large randomized experiment (*N* = 3027), we study methodological aspects of stated-preference elicitation with payment cards (price lists) in an extreme poverty context. In a 2 × 2 design, we systematically vary whether buying or selling prices are measured, crossed with the range of the payment card.

**Results:**

We find substantial effects of both the pricing method and the list range on elicited VSLY. Estimates of the gross domestic product per capita multiplier for VSLY range from 3.5 to 33.5 depending on the study design. Importantly, all estimates are economically and statistically significantly larger than the current World Health Organization threshold of 3.0 for cost-effectiveness analyses.

**Conclusions:**

Our results inform design choice in VSLY measurements, and provide insight into the potential variability of these measurements and possibly robustness checks.

## Introduction

The value of a statistical life year (VSLY) provides an economic measure of a decision maker’s personal trade-off between the risk of death and other consumption. It is a core input to policy analysis for risk reductions [1, 2]. Because it is based on how those people affected by some risk value a marginal reduction in the risk, its use avoids undue paternalism. By measuring VSLY for a group of people, averaging their VSLY provides a benchmark that can be used in policy assessments relevant for that group, without the need to measure its value repeatedly for each policy question. That is, the average population (of interest) VSLY forms an important policy input for cost–benefit analysis of risk reduction regulation and investment.

In the health domain, cost-effectiveness analysis similarly assesses the efficiency of health interventions by comparing their monetary costs to the benefits in terms of lives saved. Similar to risk regulation, a population benchmark for those affected is typically applied, in particular measured as a multiple of the relevant per capita gross domestic product (GDP). The World Health Organization defines an intervention as (highly) cost effective if it costs up to three times GDP per live year saved [3, 4], based on expert consensus about the value of economic productivity as well as the additional value of leisure time, market consumption, pure longevity effects, and the value of pain and suffering attributable to disease [5]. It has been argued, however, that the GDP multiple of 3 is not based on any empirical assessment [6] and that, especially in low-income countries, the benefits as perceived by the population exposed to health risk may be much larger than this benchmark [7]. Because extrapolation of VSLY estimates from rich countries with good data availability to low-income countries is difficult, and depends on still poorly understood estimates of income elasticities of VSLY [8], direct measurements in low-income contexts are warranted. Because of the lack of detailed data on job-related mortality risks and the resulting job wage differentials outside the most advanced economies, such measurements must be based on stated-preference techniques (even for many advanced economies).

Unfortunately, very few such measurements in low-income countries exist [9] (see also Robinson and coauthors [2], for a recent survey). In settings of extreme poverty, illiteracy and cultural context often complicate the elicitation of valuations, and this is especially true in the context of complicated life risks [7]. Developing and testing protocols that can be widely applied in low-income countries is therefore of foremost importance. The current study contributes to this research agenda by (1) addressing methodological questions with regard to the pricing of life risks in stated preference surveys, and by (2) providing empirical estimates on VSLY in an extreme poverty region, using a large randomized experiment. In particular, we study the sensitivity of popular choice-based payment card (or price list) methods [10–12] to several design aspects. Price list methods are often used to measure individual-level indifference values. In contrast to single-choice methods that potentially provide too little data, and open-ended questions that may be hard to answer, choice-based price list procedures allow participants to weigh costs and benefits and to approach their valuation as prices increase sequentially. However, we argue that these measurements may be sensitive to problems identified in the behavioral decision-making literature, and which have so far exclusively been studied in the context of cost–benefit analyses and VSLY measurements in high-income settings [13].^1^

First, choices may either be framed in terms of willingness-to-pay (WTP) assessments, i.e., as buying price for reduced risk, or alternatively in terms of willingness-to-accept (WTA), i.e., as selling price for accepting increased risk. Often, the choice of WTP or WTA may be dictated by the nature of the risk transformation, e.g., increased health risks due to the placement of a risky factory site (thus WTA). However, for general purpose measurements, it is a-priori unclear which of the two measures is the “correct” way to measure monetary valuations of risk changes [14]. It is common to employ WTP when measuring the economic impact of health-related issues. The Guidelines for Preparing Economic Analyses by the US environmental protection agency, page 94, suggest that “Willingness to pay to reduce the risk of experiencing an illness is the preferred measure of value for morbidity effects.” [15] However, for many risks to which people become exposed it is not clear that WTP is the obvious choice (e.g., Bishop and coauthors measure of damages due to the BP oil spill based on WTP [16]). Unfortunately, the decision between WTP and WTA is not harmless. A large body of stated-preference literature has documented a substantial discrepancy between valuations based on WTP and WTA, although in theory only a small gap can be rationalized, for instance due to wealth effects. Horowitz and McConnell reviewed roughly 200 studies and found the average ratio of WTA to WTP is about 7.2, with a median of 2.6 [17]. Policy recommendations based on an alternative framing may therefore have strong ramifications for policy decisions.

Second, payment card methods elicit indifference points for risk changes through a list of ordered binary choices for different prices (“price list”). In particular, participants are presented with a set of potential prices, typically in increasing order. For each price, they have to choose whether they are willing to pay this amount for a defined risk reduction (respectively accept the price for a risk increase in WTA). This choice list method allows identifying, at the individual level, the price for which participant is indifferent between buying (respectively selling), and accepting the risk. The price list method is commonly employed in the contingent valuation literature (cf. the paper by Neumann‑Böhme and coauthors [18]). While price lists are a useful decision analytic tool helping respondents to identify their valuation, the selection of the potential prices on the list may influence their decisions. The range and the step size of prices on the list may serve as reference points in the respondents’ assessment of the risk [19–21]. Again, within a range of potentially reasonable values, it is not clear what would be the “correct” range and step size of the prices on the list.

Arguing for the importance of more direct measurements of VSLY in low-income countries, Patenaude and coauthors provide a design and method for measuring stated-preference based VSLY in low-income and low-literacy contexts [7]. They carefully tailor the elicitation procedure towards the relevant pool of respondents in low-income settings. The current study builds on the basic paradigm proposed by Patenaude and coauthors to address the effects of the pricing method (WTP or WTA) and the range and step size choices for price-list elicitation of VSLY [7]. In their study in Tanzania, which provides the first measurement of VSLY in sub-Saharan Africa, the authors report a multiple of 4.5 times GDP per capita, significantly larger than the World Health Organization threshold of 3 [7]. We aim to assess the robustness of this finding with respect to variation in the elicitation method. In particular, our design allows us to provide a range of per-capita GDP multiples rather than a single point measurement, that we then compare with the World Health Organization threshold.

## Methods

### Study area and population

The study area is the Nouna Health and Demographic Surveillance System (HDSS) site located in a rural area in northwestern Burkina Faso [22]. In 2015, the HDSS site covers a population of 107,000 individuals in 15,000 households. All individuals in the HDSS have a unique identifier and Geographic Information Systems (GIS) data are available for all households. Surveillance and research in the Nouna HDSS are conducted by the Centre for Research on Health in Nouna (Centre de Recherche en Santé de Nouna, CRSN).

This paper uses data from the CRSN Heidelberg Aging Study (CHAS), a cross-sectional study investigating the health of older adults in Nouna [23, 24]. CHAS used a two-stage random sample to identify 3998 individuals aged over 40 living in the HDSS site, of whom 3027 participated in the study and completed the questions on VSLY (of the 971 non-participating individuals the largest share moved away or passed away; 196 individuals did not want to participate in the study, and 112 did not give consent; 1 person participated in other parts of the study, but not in the VSLY module). Individuals were interviewed at or near their homes in the local language of their preference.

### VSLY measurement

We randomized each CHAS participant to one of the four conditions of our 2 × 2 design for the VSLY question. Because Patenaude and coauthors showed suggestive sex and age differences in VSLY [7], we stratified our randomization by sex and 10-year age brackets. Our elicitation approach closely follows the novel approach and scenario developed for application in rural and very low-income country contexts by Patenaude and coauthors [7]. We ask participants to make a hypothetical monetary judgment of the value of a hypothetical mortality risk reduction or increase (a contingent valuation). The hypothetical nature of the monetary assessment and the involved risks are made clear to the participants. It is also communicated that the monetary amount specified by the participant is a subjective judgment, and that there are no right or wrong answers to the question.

**Fig. 1:**
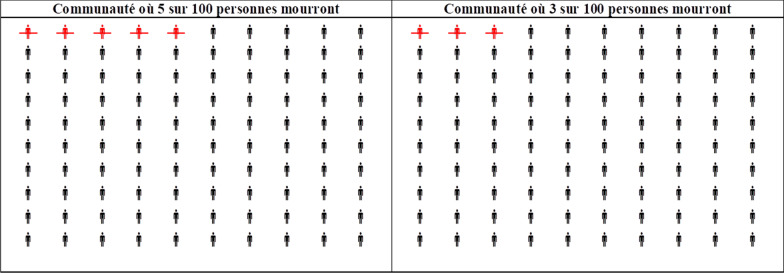
Graphical representation of health risks

More specifically, we administered a contingent valuation survey to elicit the recurring annual willingness-to-pay (resp. willingness-to-accept) for a permanent 2% reduction (resp. increase) in risk of death from a 5% to a 3% level (from a 3% to a 5% level). The 5% risk represents the typical mortality rate in Burkina Faso, as determined by life tables for 2015.^2^ Risk changes were chosen to allow meaningful communication to our mostly illiterate population (see Fig. 1). Participants were randomized to receive either a WTP or WTA question. We further factorially randomized participants to receive either a small or large range of payment amounts as response options. Both price lists start at zero CFA; the small range of payments had an upper bound of 400,000 CFA (about US$ 1993); the large range of payment had an upper bound of 2 million CFA (about US$ 9966). For comparison, gross domestic product per capita at purchasing power parity in Burkina Faso was US$1,862 in 2017, when the experiment was designed.^3^ The bounds were selected to cover the benchmark case by Patenaude and coauthors [7], which involved an upper bound of TZS10m, which was about US$ 5,000 at the time of the study. Interviewers presented choices for prices in an ascending order to the participants. Both lists contained 20 prices; thus, the average price step in the small-range list was substantially lower than in the large-range list, i.e., the latter led to larger values more quickly. A transcript of the full study material is provided in “Appendix 2”.

Interviewers described to respondents two identical communities, which only differ with regard to the probability that each of its members may suffer a sudden and painless death in each year. The different risks in the two communities were illustrated graphically using natural frequencies rather than percentage risks. A matrix showed the number of people who die each year, out of 100 people in the community (see Fig. 1). Respondents were told to imagine being one of these 100 people in one of these communities (lower-risk community for WTA; larger-risk community for WTP), without knowing whether they will be one of the majority of people who live or one of the few who die. Respondents were then asked to specify the annual price they would pay to move to the lower-risk community (WTP), respectively, the payment they require to move to the larger risk community (WTA). Language was intentionally kept neutral and unemotional when describing risks and decisions. This scenario resulted from the development of a feasible design for very low-income contexts by Patenaude et al. [7], making it an appropriate design for our population.

Following Patenaude and coauthors [7], the valuation question asks the individual to consider a recurring annual payment they would be willing to pay, for a persistent risk reduction in their remaining life. Usually, VSLY results from eliciting Value of Statistical Life (VSL) and dividing by expected life years remaining based on the age of the respondent. As in the paper by Patenaude and coauthors [7], our population concerns an extreme poverty context, so life expectancy may not be in line with national life tables. Moreover, individuals make real decisions over value and willingness to pay based on their perceived longevity, not empirical statistical average longevity in a group. This assumes individuals are better judges of their longevity and underlying heath, genetic, and other and risk factors than population average life expectancy. The value we elicit in our paper therefore is mathematically equivalent to VSL divided by undiscounted life expectancy (which is now best practice for VSLY [2]), but where life expectancy is subjectively assessed (recurring payment for life) from each individual. This allows us to calculate the VSLY directly from the questionnaire: Since the observed WTP/WTA is the value of a 2% risk reduction/increase, our VSLY is obtained by multiplying this elicited WTP/WTA measurement by 50 to get to the annual value of a 100% reduction/increase in statistical risk of death [25, 26].

### Hypotheses

Based on the behavioral decision making and preference elicitation literature we predict that (1) WTA elicits larger VSLY than WTP [19, 20, 27], and that (2) the large-range (large-step) choice lists elicit larger VSLY than the small-range (small-step) lists [12, 21, 28]. Additionally, we test the hypothesis whether the GDP-per-capita multiple of the VSLY is equal to 3.0 versus larger than 3.0—the World Health Organization (WHO) recommended multiple [7].

## Results

In this section, we report the results based on the full sample of *N* = 2907 individuals who provide valid answers based on the choice-list method. Robustness checks using either trimmed data (removing boundary values), or including non-standard answers (that were not part of the choice list) are provided in “Appendix 1.4 and 1.5” respectively. Overall, surveyors indicated that participants understood the task very well. On a scale from 1 (minimum confidence) to 10 (maximum confidence) that the participant understood the procedures properly, the modal and medium scores were 10 and 9, respectively. We conclude that, despite the complexity of mortality risk pricing, the good understanding of the task indicates that the materials developed by Patenaude and coauthors work well in low-income low-literacy contexts [7]. In “Appendix 1.5,” we give the main result for the restricted sub sample of those participants who were scored with the maximum score on this question.

### Demographics

Key demographic information is summarized in Table 1 for each of our four conditions. We have a balanced sample of males and females. Participants are on average 54 years old, 70% of them are currently married and 88% of them have children. On average, they are neither satisfied nor dissatisfied with their health. In the absence of reliable income data, respondents’ household assets are used to construct the wealth index [29]. It is calculated using polychoric principal component analysis, and validated using household expenditure and education data (Poirier and coauthors offer more details regarding the construction of the index specifically for our sample [30]).

**Table 1:** Socioeconomic and demographic summary statistics

	WTP_small	WTP_large	WTA_small	WTA_large	All
Female (%)	0.52 (0.50)	0.50 (0.50)	0.51 (0.50)	0.50 (0.50)	.51 (0.50)
Age (years)^a^	54.56 (11.01)	54.00 (11.21)	54.53 (10.77)	54.27 (10.89)	54.34 (10.97)
Currently married (%)	0.70 (0.46)	0.69 (0.46)	0.70 (0.46)	0.71 (0.45)	0.70 (0.46)
Has children (%)	0.87 (0.34)	0.86 (0.34)	0.88 (0.33)	0.89 (0.31)	0.88 (0.33)
Perceived health (1 = worst; 5 = best)	3.34 (0.97)	3.34 (0.98)	3.32 (0.99)	3.34 (0.99)	3.34 (0.98)
Wealth Index Score (− 3.7 to 5.4)	0.01 (1.44)	− 0.07 (1.32)	0.04 (1.40)	0.09 (1.42)	0.02 (1.40)
Observations	736	714	725	732	2,907

Standard deviations in parenthesis

^a^Missing age data for 3 participants (*N* = 2904). All pairwise t-tests for differences between groups are insignificant after correcting for multiple testing

### VSLY measurements

**Fig. 2:**
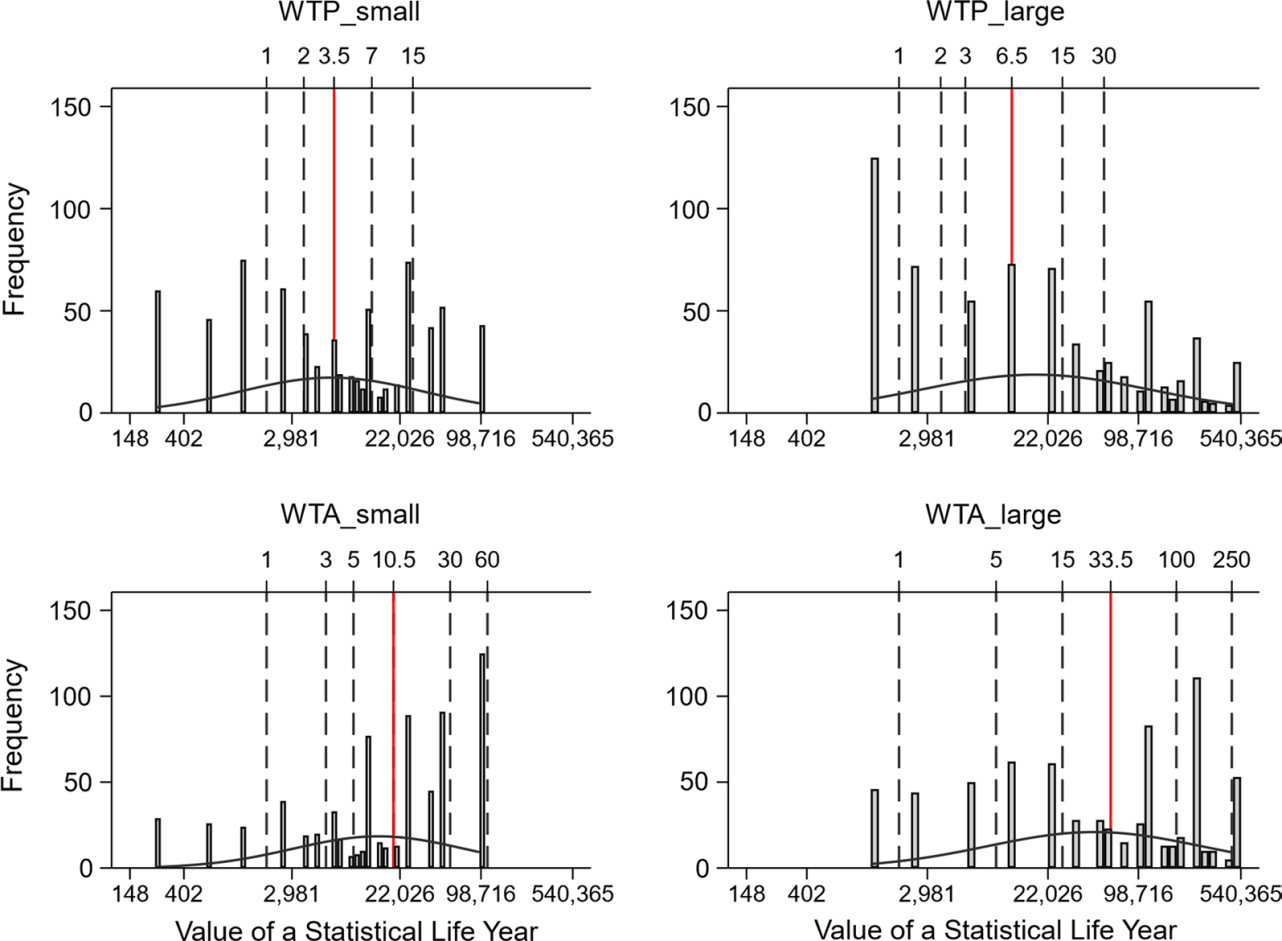
Distribution of elicited Value of a Statistical Life Year (VSLY). Notes: Value of a statistical life year (VSLY) as measured in our survey is reported in 2017 purchasing power parity (PPP)-adjusted international dollars. The VSLY in our survey are better represented by a lognormal distribution, so the scale of the x-axis (VSLY) has been log-transformed for ease of visualization and to better reflect the underlying log-normal distribution. The multiples of annual GDP per-capita income in PPP dollars are shown at the top of the x-axis and indicated by dashed vertical lines. They are derived for per capita GDP of $1862 (2017 international dollars). The solid vertical lines in red are the multiples based on median VSLY in each condition

Figure 2 presents a compact summary of the observed data, and provides insights regarding the variability of the data. The figure shows that the different methods shift the whole distribution of elicited values, but also affect the shape of the distribution of values. Detailed comparisons of WTP/WTA by condition are shown in Table 2. The upper panel shows the annual indifference payment values (labeled: Price) for a persistent 2% change in mortality risk. The middle panel shows the corresponding VSLYs, and the lower panel shows the resulting GDP-per-capital multiples based on median VSLYs. Elicited valuations differ substantially across the four conditions, with large-range price lists eliciting substantially higher values than small-range price lists, and WTA eliciting larger values than WTP. GDP per capita multiples for median VSLY range from 3.5 for a small-range WTP price list to 33.5 for large-range WTA price list. Compared to the small-range WTP price list benchmark, the change in median VSLY is larger when moving to WTA, while the change in means is larger when moving to the large-range price list. That is, larger ranges in the list lead to more skewed values. All four GDP per capita multiples are significantly larger than the WHO cost effectiveness threshold of 3.0.

**Table 2:** Indifference values, VSLYs, and GDP multiple across conditions

	WTP_small	WTP_large	WTA_small	WTA_large
Price: Mean	351	1394	647	2491
Price: Median	125	249	399	1246
Price: 95% CI	74–175	11–488	332–466	960–1531
VSLY: Mean	17,526	69,724	32,343	124,526
VSLY: Median	6229	12,457	19,931	62,285
VSLY: 95% CI	3716–8742	527–24,387	16,581–23,281	47,998–76,572
GDP multiple (ratio of VSLY median to per capita income), tested against WHO recom-mendation^a^	3.5 (*p* val = 0.036)	6.5 (*p* val < 0.001)	10.5 (*p* val < 0.001)	33.5 (*p* val < 0.001)
Observations	736	714	725	732

WTP_small indicates WTP elicitation using a small-range price list, etc. Entries in PPP adjusted 2017 international $. Price stands for the amount respondents are willing to pay or receive on an annual basis. The 95% Confidence Interval is constructed around the median, using the standard built-in procedure (the *centile* command) in Stata. The GDP per capita annual income in PPP dollar in Burkina Faso is $1,862 in 2017

^a^The median VSLY is tested against the WHO-recommended three times per capita income, non-parametric two-sided sign tests

We next present a multivariate analysis of the pooled data over all four conditions (raw correlations are shown in Table 4 in “Appendix”). We conduct linear regression analysis of four different models, shown in Table 3. In the simple treatment comparison (Model 1) both large-range and WTA significantly increase VSLY, and there is a significant interaction between the two. Adding wealth (Model 2) does not alter these results, and wealth and VSLY are positively associated, consistent with previous findings. Adding gender, age, and marital status (Model 3), and additionally self-reported health perception and cigarette expenditure as a measure of risk attitude (Model 4) does not qualitatively or quantitatively change the treatment results, although wealth is no longer significantly associated with VSLY after adding the other sociodemographic variables. In models 3 and 4, VSLY is negatively associated with being female; in Model 4 VSLY is negatively associated with higher cigarette expenditures. For US labor market-based data, Viscusi and Hersch also find lower VSLY for women, but no differences between smokers and non-smokers [31]. Table 5 in “Appendix” provides further details on how individual characteristics influence VSLY respectively in each treatment.

**Table 3:** Determinants of VSLY

VSLY	(1)	(2)	(3)	(4)
Large range	52,198*** (4384)	52,410*** (4388)	51,768*** (4369)	51,843*** (5602)
WTA	14,817*** (1577)	14,729*** (1585)	14,433*** (1570)	12,770*** (2234)
Interaction of WTA and Large range	39,984*** (6990)	39,649*** (6977)	40,323*** (6945)	29,933*** (8915)
Wealth Index		2804** (1274)	1714 (1312)	2047 (1667)
Female			− 15,466*** (3545)	− 15,584*** (4613)
Age			− 347.1** (151.4)	− 212.1 (222.4)
Married			6890* (3762)	8229 (5105)
Self-reported health (1–5 scale)				− 4,301 (2,650)
Cigarette expenditure				− 2442*** (724)
Constant	17,526*** (924.3)	17,497*** (932.3)	39,701*** (9437)	47,517** (19,188)
Observations	2907	2907	2904	1554
R-squared	0.163	0.164	0.176	0.164

Ordinary least squares (OLS) regression results are presented. The outcome is VSLY measured in PPP adjusted 2017 international $. Large Range is a dummy variable that equals to 1 if the covered range of the choice list is large. WTA is a dummy variable that equals to 1 if the choice list concerns payments to accept an increased risk. Wealth index is calculated at the household level, but vast majority of households have only one family member interviewed. Age is age in years, female is a binary indicator for female, and married is a binary indicator for being currently married. Self-reported health is measured on a 1–5 Likert Scale with 5 being the highest health status and 1 being the lowest. Cigarette expenditure is measured by the self-reported total household expenditure on cigarette in the past 7 days in PPP adjusted 2017 international $; measured only for half of the participants. Robust standard errors in parentheses: ***p < 0.01, **p < 0.05, *p < 0.1

An important question in VSLY measurement concerns the validity of the estimates, i.e., that participants comprehend the question and carefully consider their answers. We have provided robustness checks with respect to extreme answers, and have shown that demographics predict valuations in a sensible way. In an additional analysis, we focus on the effect of the choice list method more directly. In “Appendix 1.3”, we replicate the analysis of Table 3, but use the indifference point (i.e., step-level in the choice list) as a dependent variable. Note that, although valuations monotonically increase with the indifference point, each step-level is associated with a larger valuation in the large-range compared to the small-range lists. That is, sensitivity to the economic content of the valuation tasks implies that participants should be indifferent at earlier (lower) steps in the large-range lists. Table 6 shows that this is exactly what we find (while replicating the other patterns observed in Table 3). There is an economically and statistically significant und substantial negative effect of large range on the indifference point. That is, participants react to the economic content of the lists and the scope of values. Still, as we have seen, in terms of valuations they do not adjust their behavior sufficiently, being influenced by the framing of the task.

## Discussion

Given the lack of data on VSLY for low income countries, and the difficulty to project estimates from high income countries, more direct evidence from low income settings is warranted [1, 7, 8]. This is especially true in contexts where income increases rapidly due to progress in development [32]. Patenaude and coauthors provide tools for such direct measurement in low income countries with a special focus on sub-Saharan Africa, and argue that current standards for cost-effectiveness assessments may be too low for these regions [7]. Their study focuses on how to implement VSLY elicitation in poor and illiterate populations. We build on their approach to address a problem of broad interest in the context of pricing lives in low-income context, namely the influence of different design aspects on the estimated GDP multiple. Hypotheses are derived from the literature on contingent valuation and behavioral decision making [33–36]. Our results show that the measurement of VSLY is indeed highly sensitive to the elicitation method: the elicited GDP multiples are found to differ by a factor 10 between the smallest and largest estimate.

Given the large variability of the estimates obtained, applied researchers may ask which approach is the correct one. Unfortunately, there is no easy answer to the question what the correct elicitation design is. For the question of whether WTP or WTA is more appropriate, we may distinguish between paying for improvements, thus WTP, versus accepting deterioration, thus WTA, in health risks [27]. However, Knetsch and coauthors observe that people evaluate outcomes in comparison to an internally construed reference state [37]. If people are not highly aware of mortality risk, their reference state may simply be “to be alive.” Any mortality risk may then be located in the loss domain, and WTA may be the better measure give a reference-dependent utility framework [38]. The lower WTP-based VSLY measures may then result in underinvestment in health interventions. Irrespective of these considerations, the WHO convention of using three times per capita GDP as the benchmark for cost-effectiveness is clearly too low given our estimates for either WTP or WTA.

Price list methods are often used if researchers want to measure individual-level indifference values. Single-choice methods do not provide enough data, and open-ended questions may be very hard to answer, especially for complex issue such as mortality risks. The choice-based price list procedure allows participants to weigh costs and benefits, and to approach their valuation as prices increase sequentially. On the downside, as we have shown, this procedure can be strongly affected by the design of the list. Unfortunately, for the question of how to structure the price list (range, steps), there is no simple answer either. While it is typically impractical to expose respondents to multiple elicitation tasks to identify individual-level sensitivity to the range, randomized variation across respondents, as in our study, is often feasible. The different ranges can be chosen to cover previously reported values for similar contexts. This allows to provide population averages or medians across the pooled sample of methods used, or, as we did in the current study, to provide more direct insights into the possible range of VSLY measures by showing the full range of uncertainty.

We reported median VSLY ranging from 3.5 times to 33.5 times the GDP per capita across four treatment conditions. Restricting our attention to the WTP measurements, we find VSLY ranging from 3.5 to 6.5 in terms of GDP per capita multiples. Our sample characteristics are similar to those of Patenaude and coauthors [7] in terms of proportion of married people, individuals who have children, self-perception of health, but our sample is notably older (on average 15 years older), and we have more male participants. Despite these differences, the WTP-based VSLY value of 4.5 estimated by Patenaude and coauthors falls into the range identified in the current study [7]. Recent WTP estimates from Bangladesh using a titration method instead of price lists show GDP multiples of about 7, thus even somewhat larger than the values found in the current study [9]. Note also that, while our research was conducted pre-COVID-19, we observe that in our regression models, wealth had only modest or no statistically significant influence on elicited VSLY. As such, despite the recent COVID-19 pandemic potentially having an adverse impact on socioeconomic status and wealth in Burkina Faso, we would not expect such shocks to significantly impact the elicited range of VSLY results.

Clearly, both Patenaude and coauthors’ [7] and our sample are characterized by low literacy and extreme poverty. Elicitation of valuations for hypothetical but unpleasant events, as is the case for mortality risk, using counterfactual thinking (risk increases or reductions), is far from trivial and potentially prone to error. Although the results obtained here and in Patenaude and coauthors are not meant to generalize to western populations [7], we may still ask whether results do broadly “make sense,” or whether the setting potentially harmed the elicitation process. To this end, we can compare our results to those of established studies for the USA. Viscusi discusses recent estimates for the US based on labor market data [1]. Such data are not available in most countries, making contingent valuation studies necessary, which then suffer from the methodological issues that were the focus of the current study. Viscusi reports recent estimates of VSLY of US$ 411,000 for the US (in 2015 dollars [25]). With a GDP per capita of about US$ 57,000 in 2015, the resulting GDP-per-capita multiple would be in range of 7.21 for the USA. For an older cohort of people older than 50, a maximum value of 9.56 for the GDP multiple at age 54 can be calculated from the Aldy and Viscusi’s data [1]. A recent review of 133 estimates in the literature on VSLY found a median value of €164,000, mapping on a GDP multiple of more than 6 [39]. Thus, while the VSLY values that we elicited look large, they certainly fall within the range of values observed in other studies using different methodology and different populations. As discussed by Viscusi, people at different income levels face different trade-offs [1]. VSLYs in low-income countries may be lower than in the USA in absolute terms, but may not necessarily be lower in relative terms.

The data suggest that there may be many cost-effective interventions in low-income context which are not considered under the current policy using the threshold of three times GDP. Efficiency does not imply affordability. As the World Health Organization argues, these interventions provide good value: If low-income countries cannot afford them, the international community should find ways to support their efforts [3].

## Conclusions

For policy purposes, the important insight is that even the lowest GDP multiples elicited in our study are significantly larger than the WHO recommendation of three times the local annual income, as a measure of the benefit of health interventions. Given the sensitivity of VSLY measurement to the method employed, and the important role the results play in cost-effectiveness assessments, we recommend to employ different elicitation methods to obtain a range of VSLY when measuring it for policy purposes. We focused on range effects and WTP versus WTA. Andersson and Svensson point to additional features that may become important, notably the size of the assessed risk change [40]. If the risk change is not determined by substantial factors of the policy question at hand, it may be useful to also consider that dimension. With more studies including multiple designs, meta-analyses will be able to identify the effects of different design features to allow for more robust estimates [19, 27].

## Data Availability

Data are not publicly available as consent was not given by participants for data to be shared openly. This is in part because entire age cohorts of some villages are included in the dataset, potentially allowing for deductive disclosure with sufficient local information. For this reason, anonymized data is available from CHAS study data controllers only following signature of a data use agreement restricting onward transmission. Anyone wishing to replicate the analyses presented, or conduct further collaborative analyses using CHAS (which are welcomed and considered based on a letter of intent), should contact Dr. Guy Harling (g.harling@ucl.ac.uk) in the first instance.

## Appendix 1: additional analyses

### Correlation of variables

Table 4 provides the raw correlation of the control variables and VSLY.

**Table 4:** Correlation tables of key variables

	VSLY	Wealth Index	Female	Age	Married	Self-reported health	Cigarette expenditure
VSLY	1						
Wealth Index	0.04*	1					
Female	− 0.10***	− 0.06**	1				
Age	− 0.06***	− 0.13***	0.11***	1			
Married	0.08***	0.17***	− 0.32***	− 0.34***	1		
Self-reported health	− 0.03	0.12***	− 0.15***	− 0.28***	0.11***	1	
Cigarette expenditure	− 0.05	0.05	− 0.09***	− 0.11***	0.03	0.04	1

Pearson's linear correlation coefficient: **p* < 0.05, ***p* < 0.01, ****p* < 0.001

### VSLY determinants by treatment

It may be of interest how individual characteristics influence valuations in different treatments. Table 5 below summarizes these results.

**Table 5:** Effect of demographics by treatment

VSLY	WTP_small	WTP_large	WTA_small	WTA_large
Wealth Index	− 396.6 (834.8)	2450 (4273)	− 64.14 (1402)	5277 (4653)
Female	− 9544*** (2616)	− 15,636 (10,652)	− 7921** (3747)	− 29,129** (14,695)
Age	− 119.9 (113.8)	− 99.93 (540.1)	− 445.7** (194.8)	− 230.0 (604.9)
Married	4086* (2266)	12,299 (11,637)	3775 (4142)	11,411 (16,469)
Self-reported health (1–5 scale)	− 5372*** (1521)	− 4634 (6314)	669.9 (2037)	− 8310 (7732)
Cigarette expenditure	− 784.2** (338.8)	− 4277*** (918.0)	− 254.6 (1,284)	− 2570 (6889)
Constant	44,662*** (10,481)	92,549* (47,157)	54,832*** (16,022)	161,039*** (52,691)
Observations	402	396	358	398
R-squared	0.080	0.018	0.048	0.022

OLS regression results are presented. The outcome is VSLY measured in PPP adjusted 2017 international $. Wealth index is calculated at the household level, but vast majority of households have only one family member interviewed. Age is age in years, female is a binary indicator for female, and married is a binary indicator for being currently married. Self-reported health is measured on a 1–5 Likert Scale with 5 being the highest health status and 1 being the lowest. Cigarette expenditure is measured by the self-reported total household expenditure on cigarette in the past 7 days in local currency; measured only for half of the participants. Robust standard errors in parentheses: ****p* < 0.01, ***p* < 0.05, **p* < 0.1

### Analysis of the selected row on the price list

Instead of analyzing the monetary amount (VSLY) as in the main text, here we analyze whether and how the treatment variables affect the indifference-row emerging from the choice list procedure for each respondent. For WTP, the selected row indicates the maximum annual fee a respondent is willing to pay to live in a lower-risk community. For WTA, the selected row indicates the minimum annual compensation a respondent demands for living in a higher-risk community (Table 6).

**Table 6:** Determinants of the selected row

Selected row	(1)	(2)	(3)	(4)
Large range	− 2.94*** (0.31)	− 2.93*** (0.31)	− 2.97*** (0.31)	− 2.77*** (0.41)
WTA	3.268*** (0.34)	3.26*** (0.34)	3.24*** (0.33)	3.13*** (0.46)
Interaction of WTA and large range	− 0.547 (0.44)	− 0.56 (0.44)	− 0.52 (0.44)	− 1.06* (0.60)
Wealth Index		0.14* (0.08)	0.05 (0.08)	0.06 (0.11)
Female			− 1.07*** (0.23)	− 1.17*** (0.32)
Age			− 0.024** (0.01)	− 0.02 (0.02)
Married			0.70*** (0.26)	0.65* (0.36)
Self-reported health (1–5 scale)				− 0.38** (0.16)
Cigarette expenditure				− 0.18*** (0.06)
Constant	9.91*** (0.24)	9.91*** (0.24)	11.28*** (0.71)	12.72*** (1.27)
Observations	2907	2907	2904	1554
R-squared	0.119	0.120	0.135	0.130

OLS regression results are presented. The dependent variable is the row selected by the respondents. Large Range is a dummy variable that equals to 1 if the covered range of the choice list is large. WTA is a dummy variable that equals to 1 if the choice list concerns payments to accept an increased risk. Wealth index is calculated at the household level, but vast majority of households have only one family member interviewed. Age is age in years, female is a binary indicator for female, and married is a binary indicator for being currently married. Self-reported health is measured on a 1–5 Likert Scale with 5 being the highest health status and 1 being the lowest. Cigarette expenditure is measured by the self-reported total household expenditure on cigarette in the past 7 days in PPP adjusted 2017 international $; measured only for half of the participants. Robust standard errors in parentheses: ****p* < 0.01, ***p* < 0.05, **p* < 0.1

### Trimmed data (removing 0 s and max values)

We examine the results for VSLY with extreme values have been removed. We exclude all respondents who indicate 0 and those who indicated the largest value on the price list, because these respondents might not wish to value their lives in terms of money or are limited by the boundaries. Table 7 shows that the results in the main text are largely unaffected.

**Table 7:** Indifference values, VSLYs, and GDP multiple across conditions

	WTP_small	WTP_large	WTA_small	WTA_large
Price: Mean	262	1152	383	2004
Price: Median	125	249	249	1246
Price: 95% CI	93−157	63−435	212−287	1035−1457
VSLY: mean	13,091	57,602	19,150	100,223
VSLY: median	6,229	12,457	12,457	62,285
VSLY: 95% CI	4631−7826	3164−21,750	10,584−14,330	51,737−72,833
GDP multiple (ratio of VSLY median to per capita income), tested against WHO recom-mendation^a^	3.5 (*p* val = 0.056)	6.5 (*p* val < 0.001)	6.5 (*p* val < 0.001)	33.5 (*p* val < 0.001)
Observations	658	648	574	646

WTP_small indicates WTP elicitation using a small-range price list, etc. Entries in PPP adjusted 2017 international $. Price stands for the amount respondents are willing to pay or receive on an annual basis. The 95% Confidence Interval is constructed around the median, using the standard built-in procedure (the *centile* command) in Stata. The GDP per capita annual income in PPP dollar in Burkina Faso is $1,862 in 2017

^a^The median VSLY is tested against the WHO-recommended three times per capita income, non-parametric two-sided sign tests

### Non-standard answers and task comprehension

Tables 8 and 9 report the key tables and regressions using the full sample, including additional 120 participants who gave non-standard answers. These participants directly gave a WTP or WTA that was not part of the price list. We find that results reported in the main text are qualitatively unaffected when including these respondents.

**Table 8:** Indifference values, VSLYs, and GDP multiple across conditions

	WTP_small	WTP_large	WTA_small	WTA_large
Price: mean	360	1444	668	2473
Price: median	125	249	399	1246
Price: 95% CI	75–175	(− 35)−534	331–466	965–1526
VSLY: mean	17,981	72,196	33,415	123,655
VSLY: median	6,229	12,457	19,931	62,285
VSLY: 95% CI	3730–8728	(− 1764)−26,678	16,538–23,324	48,265–76,305
GDP multiple (ratio of VSLY median to per capita income), tested against WHO recom-mendation^a^	3.5 (*p* val = 0.014)	7 (*p* val < 0.001)	10.5 (*p* val < 0.001)	33.5 (*p* val < 0.001)
Observations	767	743	754	763

WTP_small indicates WTP elicitation using a small-range price list, etc. Entries in PPP adjusted 2017 international $. Price stands for the amount respondents are willing to pay or receive on an annual basis. The 95% Confidence Interval is constructed around the median, using the standard built-in procedure (the *centile* command) in Stata. The GDP per capita annual income in PPP dollar in Burkina Faso is $1,862 in 2017

^a^The median VSLY is tested against the WHO-recommended three times per capita income, non-parametric two-sided sign tests

**Table 9:** Determinants of VSLY

VSLY	(1)	(2)	(3)	(4)
Large range	54,215*** (5396)	54,428*** (5388)	53,794*** (5309)	56,514*** (7984)
WTA	15,434*** (1575)	15,377*** (1585)	15,153*** (1589)	13,293*** (2231)
Interaction of WTA and Large range	36,025*** (7611)	35,668*** (7586)	36,299*** (7525)	23,351** (10,638)
Wealth Index		2988** (1319)	1634 (1388)	1556 (1831)
Female			− 16,700*** (3744)	− 19,366*** (5171)
Age			− 422.3** (170.0)	− 373.9 (271.6)
Married			9256** (3698)	10,514** (4987)
Self-reported health (1–5 scale)				− 4022 (2670)
Cigarette expenditure				− 2891*** (830.8)
Constant	17,981*** (913.2)	17,960*** (921.3)	43,126*** (10,542)	56,271*** (20,722)
Observations	3027	3027	3024	1615
R-squared	0.134	0.135	0.148	0.122

OLS regression results are presented. The outcome is VSLY measured in PPP adjusted 2017 international $. Large Range is a dummy variable that equals to 1 if the covered range of the choice list is large. WTA is a dummy variable that equals to 1 if the choice list concerns payments to accept an increased risk. Wealth index is calculated at the household level, but vast majority of households have only one family member interviewed. Age is age in years, female is a binary indicator for female, and married is a binary indicator for being currently married. Self-reported health is measured on a 1–5 Likert Scale with 5 being the highest health status and 1 being the lowest. Cigarette expenditure is measured by the self-reported total household expenditure on cigarette in the past 7 days in PPP adjusted 2017 international $; measured only for half of the participants. Robust standard errors in parentheses: ****p* < 0.01, ***p* < 0.05

Table 10 reports the main analyses of Table 2 for only those participants who were scored with maximum confidence level that they understood the question properly. The table shows that the main result replicates for this subsample of participants, with strong differences between conditions as for the full sample. All conditions except *WTP_small* reveal GDP multiples significantly larger than 3.

**Table 10:** Indifference values, VSLYs, and GDP multiple across conditions (maximum confidence level that participant fully understood the question)

	WTP_small	WTP_large	WTA_small	WTA_large
Price: mean	329	1493	641	2543
Price: median	75	249	498	1246
Price: 95% CI	(− 13)–162	(− 170)–669	398–598	758–1733
VSLY: mean	16,455	74,633	32,072	127,167
VSLY: median	3737	12,457	24,914	62,285
VSLY: 95% CI	(− 639)–8113	(− 8523)–33,437	19,904–29,924	37,914–86,656
GDP multiple (ratio of VSLY median to per capita income), tested against WHO recom-mendation^a^	2.0 (*p* val = 0.03)	6.5 (*p* val = 0.02)	13.5 (*p* val < 0.001)	33.5 (*p* val < 0.001)
Observations	258	275	278	259

WTP_small indicates WTP elicitation using a small-range price list, etc. Entries in PPP adjusted 2017 international $. Price stands for the amount respondents are willing to pay or receive on an annual basis. The 95% Confidence Interval is constructed around the median, using the standard built-in procedure (the *centile* command) in Stata. The GDP per capita annual income in PPP dollar in Burkina Faso is $1862 in 2017

^a^The median VSLY is tested against the WHO-recommended three times per capita income, non-parametric two-sided sign tests

## Appendix 2: transcript of Study materials

### Variant 1 (small range, wtp)

Let me briefly explain the way I would like us to approach the following question. We have come up with hypothetical situations that we would like you to imagine yourself in. You will be faced with difficult decisions that involve the risk of death. We are not in any way suggesting that by imagining yourself in these situations, you will experience what we present you with. We are also most certainly not wishing illness or even death upon you. We simply want to find out what choices people make when they find themselves in such difficult situations. Please simply ask yourself: what would I do if I was to find myself in such a situation? There are no right or wrong answers to the question we ask. Does that make sense?

Q1. I would now ask you to imagine the following two situations. In the first situation, you live in a community in which **5 out of 100** people die every year: you are one of these 100 people, thus the chance that you will die is 5 out of 100. In the second situation, you live in a community in which **3 out of 100** people die every year: thus the chance that you will die is 3 out of 100. In both situations the death is sudden and painless. You should also imagine that except for the risk of death, everything in your life is the same in the two communities. The picture below helps to illustrate the risk. For each 100 people, the crossed-out figures will die. Imagine yourself to be one of the 100 people, but you do not yet know which one. Thus, you might be one of those who die, or one of those who do not die.

Imagine that you live in a community where the risk of death is 5 out of 100 people each year. You can now pay a certain amount of money each year to instead live in a community with a risk of death of 3 out of 100 people per year. Here we would like to know what the maximum annual fee in CFA is that you would be willing to reduce your risk of death from 5 out of 100 people to a risk of death of 3 out of 100 people. In other words, what would be the maximum annual fee in CFA that you would be willing to pay, to live in a community where the risk of death is 3 out of 100 people rather than 5 out of 100 people.

Please be sure to take into account what you can actually afford to pay, and not if you had unlimited money. Please check the box for the maximum amount of CFA below.

[Start from low amounts going up, asking “Would you pay no more than X, or more?”. If respondent indicates that his/her value is in between two values in the list, tick the two value bracketing the value indicated by respondent.]

**Table:**

	I am willing to pay this annual fee to reduce my risk of death from 5 out of 100 to 3 out of 100 per year
Amount (annual, CFA fr)	Check maximum value
0	O
1000	O
2500	O
5000	O
10,000	O
15,000	O
20,000	O
25,000	O
30,000	O
35,000	O
40,000	O
45,000	O
50,000	O
60,000	O
70,000	O
80,000	O
100,000	O
150,000	O
200,000	O
400,000	O

Q2. How confident are you that the respondent understood the questions in this section? Please indicate your impression of the respondent’s level of understanding on a scale from 0 to 10, where 0 = the respondent did not understand the question at all, and 10 = the respondent fully understood the question. Please place a circle around the number.

**Table:**

Did not understand the question at all	1	2	3	4	5	6	7	8	9	10	Fully understood the question

### Variant 2 (large range, wtp)

Let me briefly explain the way I would like us to approach the following question. We have come up with hypothetical situations that we would like you to imagine yourself in. You will be faced with difficult decisions that involve the risk of death. We are not in any way suggesting that by imagining yourself in these situations, you will experience what we present you with. We are also most certainly not wishing illness or even death upon you. We simply want to find out what choices people make when they find themselves in such difficult situations. Please simply ask yourself: what would I do if I was to find myself in such a situation? There are no right or wrong answers to the question we ask. Does that make sense?

Q1. I would now ask you to imagine the following two situations. In the first situation, you live in a community in which **5 out of 100** people die every year: you are one of these 100 people, thus the chance that you will die is 5 out of 100. In the second situation, you live in a community in which **3 out of 100** people die every year: thus the chance that you will die is 3 out of 100. In both situations the death is sudden and painless. You should also imagine that except for the risk of death, everything in your life is the same in the two communities. The picture below helps to illustrate the risk. For each 100 people, the crossed-out figures will die. Imagine yourself to be one of the 100 people, but you do not yet know which one. Thus, you might be one of those who die, or one of those who do not die.

Imagine that you live in a community where the risk of death is 5 out of 100 people each year. You can now pay a certain amount of money each year to instead live in a community with a risk of death of 3 out of 100 people per year. Here we would like to know what the maximum annual fee in CFA is that you would be willing to reduce your risk of death from 5 out of 100 people to a risk of death of 3 out of 100 people. In other words, what would be the maximum annual fee in CFA that you would be willing to pay, to live in a community where the risk of death is 3 out of 100 people rather than 5 out of 100 people.

Please be sure to take into account what you can actually afford to pay, and not if you had unlimited money. Please check the box for the maximum amount of CFA below.

[Start from low amounts going up, asking “Would you pay no more than X, or more?”. If respondent indicates that his/her value is in between two values in the list, tick the two value bracketing the value indicated by respondent.]

**Table:**

	I am willing to pay this annual fee to reduce my risk of death from 5 out of 100 to 3 out of 100 per year
Amount (annual, CFA fr)	Check maximum value
0	O
5000	O
10,000	O
25,000	O
50,000	O
100,000	O
150,000	O
200,000	O
250,000	O
300,000	O
400,000	O
500,000	O
600,000	O
700,000	O
800,000	O
1,000,000	O
1,200,000	O
1,400,000	O
1,700,000	O
2,000,000	O

Q2. How confident are you that the respondent understood the questions in this section? Please indicate your impression of the respondent’s level of understanding on a scale from 0 to 10, where 0 = the respondent did not understand the question at all, and 10 = the respondent fully understood the question. Please place a circle around the number.

**Table:**

Did not understand the question at all	1	2	3	4	5	6	7	8	9	10	Fully understood the question

### Variant 3 (small range, WTA)

Let me briefly explain the way I would like us to approach the following question. We have come up with hypothetical situations that we would like you to imagine yourself in. You will be faced with difficult decisions that involve the risk of death. We are not in any way suggesting that by imagining yourself in these situations, you will experience what we present you with. We are also most certainly not wishing illness or even death upon you. We simply want to find out what choices people make when they find themselves in such difficult situations. Please simply ask yourself: what would I do if I was to find myself in such a situation? There are no right or wrong answers to the question we ask. Does that make sense?

Q1. I would now ask you to imagine the following two situations. In the first situation, you live in a community in which **3 out of 100** people die every year: you are one of these 100 people, thus the chance that you will die is 3 out of 100. In the second situation, you live in a community in which **5 out of 100** people die every year: thus the chance that you will die is 5 out of 100. In both situations the death is sudden and painless. You should also imagine that except for the risk of death, everything in your life is the same in the two communities. The picture below helps to illustrate the risk. For each 100 people, the crossed-out figures will die. Imagine yourself to be one of the 100 people, but you do not yet know which one. Thus, you might be one of those who die, or one of those who do not die.

Imagine that you live in a community where the risk of death is 3 out of 100 people each year. You can now receive a certain amount of money each year if you accept to instead live in a community with a risk of death of 5 out of 100 people per year. Here we would like to know what the minimum annual fee in CFA is that you would require as compensation to accept your risk of death to increase from 3 out of 100 people to a risk of death of 5 out of 100 people. In other words, what would be the minimum annual fee in CFA that you would require as compensation, to accept living in a community where the risk of death is 5 out of 100 people rather than 3 out of 100 people.

Please be sure to think about the absolute minimum compensation you would require, which may not be the amount someone else is able or willing to compensate you. That is, we want to know what your personal acceptable minimum is, irrespective of what you believe could be paid by someone. Please check the box for the minimum amount of CFA below.

[Start from low amounts going up, asking “Would accept the risk increase for X, or do you require more?” If respondent indicates that his/her value is in between two values in the list, tick the two value bracketing the value indicated by respondent.]

**Table:**

	I require at least this annual fee to accept an increase in my risk of death from 3 out of 100 to 5 out of 100 per year
Amount (annual, CFA fr)	Check minimum value
0	O
1000	O
2500	O
5000	O
10,000	O
15,000	O
20,000	O
25,000	O
30,000	O
35,000	O
40,000	O
45,000	O
50,000	O
60,000	O
70,000	O
80,000	O
100,000	O
150,000	O
200,000	O
400,000	O

Q2. How confident are you that the respondent understood the questions in this section? Please indicate your impression of the respondent’s level of understanding on a scale from 0 to 10, where 0 = the respondent did not understand the question at all, and 10 = the respondent fully understood the question. Please place a circle around the number.

**Table:**

Did not understand the question at all	1	2	3	4	5	6	7	8	9	10	Fully understood the question

### Variant 4 (large range, wta)

Let me briefly explain the way I would like us to approach the following question. We have come up with hypothetical situations that we would like you to imagine yourself in. You will be faced with difficult decisions that involve the risk of death. We are not in any way suggesting that by imagining yourself in these situations, you will experience what we present you with. We are also most certainly not wishing illness or even death upon you. We simply want to find out what choices people make when they find themselves in such difficult situations. Please simply ask yourself: what would I do if I was to find myself in such a situation? There are no right or wrong answers to the question we ask. Does that make sense?

Q1. I would now ask you to imagine the following two situations. In the first situation, you live in a community in which **3 out of 100** people die every year: you are one of these 100 people, thus the chance that you will die is 3 out of 100. In the second situation, you live in a community in which **5 out of 100** people die every year: thus the chance that you will die is 5 out of 100. In both situations the death is sudden and painless. You should also imagine that except for the risk of death, everything in your life is the same in the two communities. The picture below helps to illustrate the risk. For each 100 people, the crossed-out figures will die. Imagine yourself to be one of the 100 people, but you do not yet know which one. Thus, you might be one of those who die, or one of those who do not die.

Imagine that you live in a community where the risk of death is 3 out of 100 people each year. You can now receive a certain amount of money each year if you accept to instead live in a community with a risk of death of 5 out of 100 people per year. Here we would like to know what the minimum annual fee in CFA is that you would require as compensation to accept your risk of death to increase from 3 out of 100 people to a risk of death of 5 out of 100 people. In other words, what would be the minimum annual fee in CFA that you would require as compensation, to accept living in a community where the risk of death is 5 out of 100 people rather than 3 out of 100 people.

Please be sure to think about the absolute minimum compensation you would require, which may not be the amount someone else is able or willing to compensate you. That is, we want to know what your personal acceptable minimum is, irrespective of what you believe could be paid by someone. Please check the box for the minimum amount of CFA below.

[Start from low amounts going up, asking “Would accept the risk increase for X, or do you require more?” If respondent indicates that his/her value is in between two values in the list, tick the two value bracketing the value indicated by respondent.]

**Table:**

	I require at least this annual fee to accept an increase in my risk of death from 3 out of 100 to 5 out of 100 per year
Amount (annual, CFA fr)	Check minimum value
0	O
5000	O
10,000	O
25,000	O
50,000	O
100,000	O
150,000	O
200,000	O
250,000	O
300,000	O
400,000	O
500,000	O
600,000	O
700,000	O
800,000	O
1,000,000	O
1,200,000	O
1,400,000	O
1,700,000	O
2,000,000	O

Q2. How confident are you that the respondent understood the questions in this section? Please indicate your impression of the respondent’s level of understanding on a scale from 1 to 10, where 1 = the respondent did not understand the question at all, and 10 = the respondent fully understood the question. Please place a circle around the number.

**Table:**

Did not understand the question at all	1	2	3	4	5	6	7	8	9	10	Fully understood the question

## Abbreviations

CERS: Comité d’Ethique pour la Recherche en Santé
CFA: Franc of the Financial Community of Africa
CHAS: CRSN Heidelberg Aging Study
CIE: Institutional Ethics Committee
CRSN: Centre de Recherche en Santé de Nouna
GDP: Gross domestic product
GIS: Geographic information system
HDSS: Health and demographic surveillance system
OLS: Ordinary least squares
PPP: Purchasing power parity
VSL: Value of statistical life
VSLY: Value of statistical life year
WHO: World Health Organization
WTA: Willingness-to-accept
WTP: Willingness-to-pay

## Footnotes

1 Payment scales are closely related to payment cards, having the respondents choose one price from a list of prices, rather than sequentially presenting choices between accepting and not accepting each price. The therefore potentially suffer from similar problems as those discussed in the current study for payment cards.

2 See the World Health Organization webpage [41].

3 See World Bank Data [42].
